# Prediction of financial deficits of postoperative patients in the intensive care unit using machine learning

**DOI:** 10.1186/s40981-025-00819-3

**Published:** 2025-10-21

**Authors:** Saori Ikumi, Takuya Shiga, Eichi Takaya, Shinya Sonobe, Yu Kaiho, Yukiko Ito, Masanori Yamauchi

**Affiliations:** 1 Department of Anesthesiology and Perioperative Medicine, Tohoku University Graduate School of Medicine, 1-1, Seiryo-machi, Aoba-ku, Sendai, Miyagi 980-8574 Japan; 2 AI Lab, Tohoku University Hospital, 1-1, Seiryo-machi, Aoba-ku, Sendai, Miyagi 980-8574 Japan; 3 Experience Design and Alliance Section, Tohoku University Hospital, 1-1, Seiryo-machi, Aoba-ku, Sendai, Miyagi 980-8574 Japan; 4 Department of Translational Neuroscience, Graduate School of Biomedical Engineering, Tohoku University, 1-1, Seiryo-machi, Aoba-ku, Sendai, Miyagi 980-8574 Japan; 5 Graduate School of Business and Commerce, Keio University, 2-14-45, Mita, Minato-ku, Tokyo, 108-8345 Japan

**Keywords:** Intensive care unit, Machine learning, Financial deficit, Postoperative patients, Insurance claims data, Contribution margin

## Abstract

**Background:**

Operational loss, defined as unanticipated financial deficits in intensive care unit (ICU) management, is challenging to predict yet critical for hospital sustainability. This study aimed to evaluate whether machine-learning models can predict financial loss events in postoperative ICU patients.

**Methods:**

We conducted a retrospective analysis of postoperative patients admitted to the ICU at Tohoku University Hospital between April 2017 and March 2021. A total of 22 clinical and administrative variables collected within 24 h of ICU admission were used to develop machine-learning models. The outcome was defined as financial loss events, determined by a negative contribution margin below the break-even threshold of − 909 USD. The dataset was randomly split into training (70%) and test (30%) sets. Predictive performance was assessed using the area under the receiver operating characteristic curve (AUC) and accuracy.

**Results:**

Among 6743 postoperative ICU patients, 425 (6.3%) experienced financial loss events. The random forest classifier demonstrated high predictive performance, with an AUC of 0.859 and accuracy of 0.785.

**Conclusions:**

Machine-learning models may accurately predict financial loss events in postoperative ICU patients, potentially supporting efficient resource allocation and hospital financial planning.

**Supplementary Information:**

The online version contains supplementary material available at 10.1186/s40981-025-00819-3.

## Background

With hospital management facing rising demands and limited resources, the efficiency and financial sustainability of healthcare systems have become increasingly important [1]. In the intensive care unit (ICU), where high-intensity treatment is provided regardless of diagnostic uniformity, the occurrence of financial deficits per patient is not uncommon and poses a challenge to hospital management. Early identification of patients likely to incur significant financial losses could inform clinical decision-making, guide the allocation of medical resources, and help mitigate institutional financial risks [2, 3].

In Japan, hospitalization costs account for 38.1% of national healthcare expenditure in 2019, with surgical admissions comprising 17.3% of these inpatient costs in 2021 (Ministry of Health, Labour and Welfare of Japan, accessed September 16, 2025: https://www.mhlw.go.jp/toukei/saikin/hw/k-iryohi/19/dl/data.pdf, https://www.mhlw.go.jp/toukei/saikin/hw/sinryo/tyosa21/dl/ika.pdf). Most tertiary emergency hospitals (97.4%) operate under a diagnosis procedure combination (DPC)-based per-diem payment system, a Japanese variant of diagnosis-related group (DRG) reimbursement [4]. Under this system, per-diem payment rates are designed by the public insurance reimbursement scheme to cover standard clinical procedures. Only a designated number of services per day are reimbursed under the DPC scheme. Consequently, some services required for patients in the ICU (i.e., the estimated sum of variable costs per service) may exceed the designated service fees covered by the DPC payment. Even when additional life-saving procedures are essential in the ICU, variable costs may surpass reimbursed revenue, resulting in a negative contribution margin—defined as revenue minus variable costs per patient [5–7]. A negative margin implies a financial loss at the individual patient level and can adversely affect the overall operational performance of hospitals. Although most postoperative patients in the ICU fall near the break-even point due to the average-based nature of under DPC reimbursements, substantial deficits do occur for some patients. Proactively identifying such patients is essential for maintaining sustainable hospital operations.

The Sequential Organ Failure Assessment (SOFA) score is a widely used severity index in critical care, incorporating six organ systems to quantify physiological derangement [8]. Since April 2018, the SOFA score has been mandatory in the Japanese DPC reporting scheme for patients in the ICU. Reportedly, the SOFA score better reflects physiological severity, compared with diagnosis codes alone [9, 10]. However, its predictive utility for economic outcomes, such as contribution margins in postoperative patients in the ICU, remains largely unexplored.

Recently, machine-learning techniques have shown promise in predicting clinical outcomes, such as ICU mortality. However, their application to cost-related outcomes, especially contribution margins, has been limited [11].

Therefore, this study aimed to develop a machine-learning-based model to predict financial loss events—defined by negative contribution margins—among postoperative patients in the ICU. We further sought to identify key clinical features contributing to these predictions, with a particular focus on the SOFA score, as an initial severity indicator of illness, and other clinical characteristics of patients.

## Methods

### Study design and data source

This retrospective, observational study analyzed data from the DPC system and electronic medical records of patients aged ≥ 16 years, who were admitted to the ICU of the Tohoku University Hospital between April 2017 and March 2021. Although SOFA score reporting became mandatory nationally in April 2018, Tohoku University Hospital began routine SOFA score entry for ICU patients in April 2017 in preparation for this change; therefore, patients from April 2017 onward were included. Postsurgical patients admitted to the ICU under the DPC per-diem payment system were eligible for inclusion. As of April 2022, the DPC scheme had been adopted by 1764 hospitals, covering approximately 480,000 calculated beds and accounting for 85% of acute care inpatient wards in Japan. All 82 university hospitals that provide advanced medical care are required to adopt the DPC system, whereas community hospitals may participate voluntarily. The DPC dataset includes patient-specific information, such as age, sex, functional status, treatments, diagnoses (coded using the International Classification of Diseases, 10th Revision), type of admission, and daily procedures (recorded using Japanese medical procedure codes). Reimbursement under the DPC scheme follows a DRG-based structure. The dataset also contains the estimated total variable costs per patient, derived from itemized pricing of surgical, laboratory, and other inpatient medical services.

### Data collection and definitions

To develop the prediction models, data on 22 input variables were extracted from the database within 24 h of ICU admission. These variables included (1) monitoring and treatment procedures: electrocardiogram monitoring, infusion pump system, intra-arterial blood pressure, central venous catheter, invasive ventilatory support, blood transfusion therapy, pulmonary artery catheter monitoring, continuous hemodiafiltration, percutaneous cardiopulmonary support, ventricular assist device, and intracranial pressure monitoring; (2) patient self-care abilities and functional status: tossing and turning (i.e., ability to turn over), independent mobility, toothbrushing, eating, changing of clothes, understanding medical examination instructions, and performing dangerous action; and (3) SOFA score components: respiration, coagulation, liver, cardiovascular, central nervous system, and renal function (see Additional file 1: Table 1). The outcome variable was whether the estimated contribution margin for postoperative patients in the ICU fell above or below the break-even point. The estimated contribution margins were calculated for each patient from ICU admission until ICU discharge. The break-even point was defined as − 909 US dollars (USD), reflecting the ICU management fee per patient-day under the Japanese public insurance system, which is approximately 909 USD (equivalent to 100,000 Japanese yen (JPY)). The break-even threshold was uniquely defined in this study based on the standard ICU management fee in Japan, whereas previous studies have used contribution margin as an outcome measure [5–7]. This amount was derived from the 2017 per-diem ICU management fee of 96,970 JPY, which we rounded up to 100,000 JPY as a threshold for defining a large financial deficit. Contribution margin was calculated as net revenue minus direct variable medical costs, where net revenue refers to the actual reimbursement received. We excluded fixed costs from this analysis, as they are typically high and not directly attributable to individual patient care. Variable medical costs included procedures, drugs, and hospital fees but excluded service fees for meals, transportation, and family support. Reimbursement was based on either comprehensive DRG-based bundled payments or additional item-by-item points. The “estimated contribution margin” was defined as total reimbursement minus direct medical costs. Currency conversion was performed using an average exchange rate of 110 JPY to 1 USD, based on the average rate between 2017 and 2021.

### Development and validation of models

The primary outcome variable was whether there was a large negative contribution margin. Prediction models were developed using random forest (RF), eXtreme Gradient Boosting (XGBoost), and support vector machine (SVM). Patients with missing or unclear SOFA scores were excluded. After the exclusion of missing or unknown data, the data were randomly split into training (70%) and test (30%) datasets. To address class imbalance, random under-sampling was applied to the training dataset at a 1:1 ratio, retaining all patients with financial loss events and randomly selecting an equal number of patients without events [12]. The balanced dataset was then used for model training, with under-sampling performed prior to applying ensemble methods. The test dataset was kept unchanged to allow unbiased evaluation. External validation using data from other institutions was not performed in this study, as the analysis was based on a single-center dataset. RF and XGBoost are ensemble learning methods based on decision trees and are known for their improved accuracy in nonlinear settings [13]. Both methods are robust in handling diverse types of data, including categorical and numerical features, making them well suited for clinical prediction tasks [14]. In contrast, SVM is a non-decision-tree-based classifier that constructs hyperplanes to separate classes in high-dimensional space [15, 16]. The RF classifier avoids overfitting and stratifies samples by considering complex interactions between variables, and it is particularly effective in high-dimensional, small-sample scenarios [17–19]. XGBoost, on the other hand, leverages gradient boosting to optimize performance through iterative improvements and often demonstrates competitive accuracy in structured healthcare datasets [13].

All models were implemented in R version 4.0.2 (R Foundation for Statistical Computing, Vienna, Austria), using the randomForest, xgboost, and e1071 packages. Hyperparameter tuning was not performed in this study. Instead, we applied the default settings of each algorithm, which we explicitly reported to ensure reproducibility. For random forest, we used 500 trees, √(number of predictors) for mtry, and a node size of 1. XGBoost settings included eta = 0.3, max_depth = 6, subsample = 1, and 100 boosting rounds. For SVM, we applied a radial kernel with C-classification and gamma set to the inverse of the number of predictors.

### Statistical analysis

Continuous variables were presented as means and standard deviations or medians and interquartile ranges, whereas categorical variables were reported as counts and percentages. To compare baseline categorical variables between the training and test datasets, we performed *χ*^2^ tests or Fisher’s exact tests, as appropriate. Continuous variables were compared using the Mann–Whitney *U* test. A *p* value < 0.05 was considered statistically significant. After the machine-learning models were trained using the training dataset, their predictive performance was evaluated on the test dataset. Model performance was assessed using the receiver operating characteristic (ROC) curve, the area under the ROC curve (AUC), accuracy, precision, recall, and F1 score. A probability threshold of 0.5 was applied to classify patients as positive or negative cases. As the RF model demonstrated superior predictive performance, feature importance analysis was conducted for this model. Variable importance was calculated using the importance function in the randomForest package, based on the mean decrease in Gini impurity. This metric reflects how much each variable contributes to the decrease in node impurity across the trees in the forest.

### Sensitivity analysis

In the sensitivity analysis, patients with missing or unclear SOFA scores were retained in the dataset. Missing SOFA values were imputed using mean imputation, with the mean calculated separately within the training dataset to avoid data leakage. The same imputation strategy was then applied to the test dataset. After imputation, model development and evaluation were performed using the same procedure as in the main analysis.

### Data access statement

The data used in this study were accessed for research purposes on August 30, 2021, and the final data analysis was completed on August 14, 2025.

### Code availability

The scripts are available from the corresponding author upon reasonable request, in line with institutional policies and data use agreements.

## Results

### Baseline characteristics

**Fig. 1:**
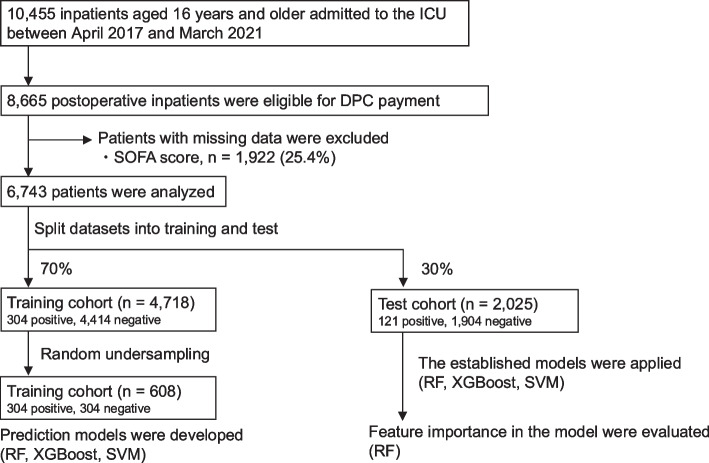
Flowchart of patient selection and machine-learning algorithm development and evaluation. *ICU*, intensive care unit; *DPC*, diagnosis procedure combination; *SOFA*, Sequential Organ Failure Assessment; *RF*, random forest; *SVM*, support vector machine

In total, 10,455 inpatients were admitted to the ICU at Tohoku University Hospital between April 2017 and March 2021. Of these, 8665 postsurgical patients admitted under the DPC scheme (per-diem payment) were eligible for inclusion. Among these, 1922 patients (25.4%) with missing or unclear SOFA scores were excluded. Finally, 6743 patients were included in the analysis (Fig. 1).

**Fig. 2:**
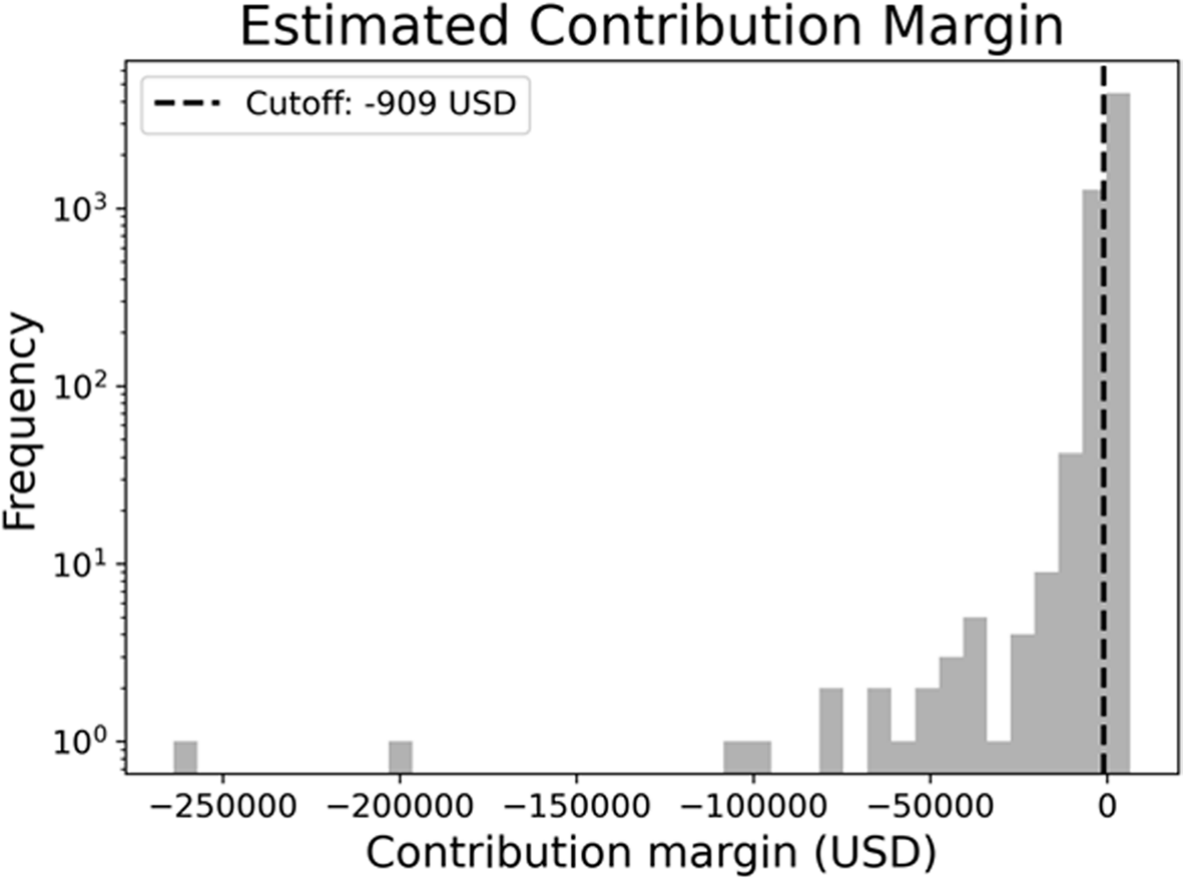
Histogram of estimated contribution margin for postsurgical patients admitted to the intensive care unit. The cutoff for large negative contribution margin was defined as − 909 USD (equivalent to 100,000 JPY). *USD*, US dollar; *JPY*, Japanese yen

The baseline characteristics of these patients are presented in Table 1. Emergency surgery was performed in 1054 (15.6%) patients. The area of surgery was cardiovascular, respiratory, abdominal, and other in 774 (11.4%), 725 (10.7%), 2132 (31.6%), and 3112 (46.2%) patients, respectively; 920 (13.6%) patients required invasive ventilatory support. The median SOFA score was 3 points. The ICU mortality rate was 0.6% (*n* = 40). The median length of ICU stay was 1 day. Median total medical costs, reimbursement, and estimated contribution margin from ICU admission to the day of ICU discharge were 1149 (interquartile range (IQR), 402–2170), 1126 (IQR, 343–2144), and − 58 (IQR, − 211–73) USD, respectively. The estimated contribution margin for most patients clustered around the median (− 58 USD); however, 425 (6.3%) patients had a contribution margin below the defined break-even point (− 909 USD), and they were classified as having a large negative contribution margin (Fig. 2).

**Table 1:** Baseline characteristics and outcomes of patients in the dataset

	Overall	Training cohort (before undersampling)	Test cohort	*p* value
Number of patients, *n*	6743	4718	2025	
Age (median (IQR))	64 (48–72)	64 (48–73)	64 (48–72)	0.138
Men, *n* (%)	3825 (56.7%)	2719 (57.6%)	1106 (54.6%)	0.024
Emergency surgery, *n* (%)	1054 (15.6%)	740 (15.7%)	314 (15.5%)	0.882
Area of surgery, *n* (%)				
Cardiovascular	774 (11.4%)	531 (11.3%)	243 (12.0%)	0.402
Respiratory	725 (10.7%)	515 (10.9%)	210 (10.4%)	0.536
Abdominal	2132 (31.6%)	1517 (32.2%)	615 (30.4%)	0.157
Others	3112 (46.2%)	2155 (45.7%)	957 (47.3%)	0.243
SOFA score (median (IQR))	3 (1–5)	3 (1–4)	3 (1–5)	0.079
Clinical outcome				0.486
Death, *n* (%)	40 (0.6%)	30 (0.6%)	10 (0.5%)	
Survival, *n* (%)	6703 (99.4%)	4688 (99.3%)	2015 (99.5%)	
Required mechanical ventilator supports, *n* (%)	920 (13.6%)	643 (13.6%)	277 (13.7%)	0.987
Length of stay in ICU (median (IQR))	1 (1–2)	1 (1–2)	1 (1–2)	0.827
Financial loss events, *n* (%)	425 (6.3%)	304 (6.4%)	121 (6.0%)	0.503
Total costs, USD (median (IQR))	1149 (402, 2170)	1154 (400, 2170)	1145 (405, 2198)	
Reimbursement, USD (median (IQR))	1126 (343, 2144)	1160 (341, 2148)	1098 (344, 2142)	
Estimated contribution margin, USD (median (IQR))	− 58 (− 211 to 73)	− 59 (− 206 to 74)	− 57 (− 221 to 72)	

Training cohort reflects the cohort prior to the random undersampling. Data are expressed as medians (interquartile ranges) for continuous variables and as exact numbers (%) for categorical variables

*IQR*, interquartile range; *ICU*, intensive care unit; *SOFA*, Sequential Organ Failure Assessment; *USD*, US dollar

### Prediction of the large negative contribution margin

**Fig. 3:**
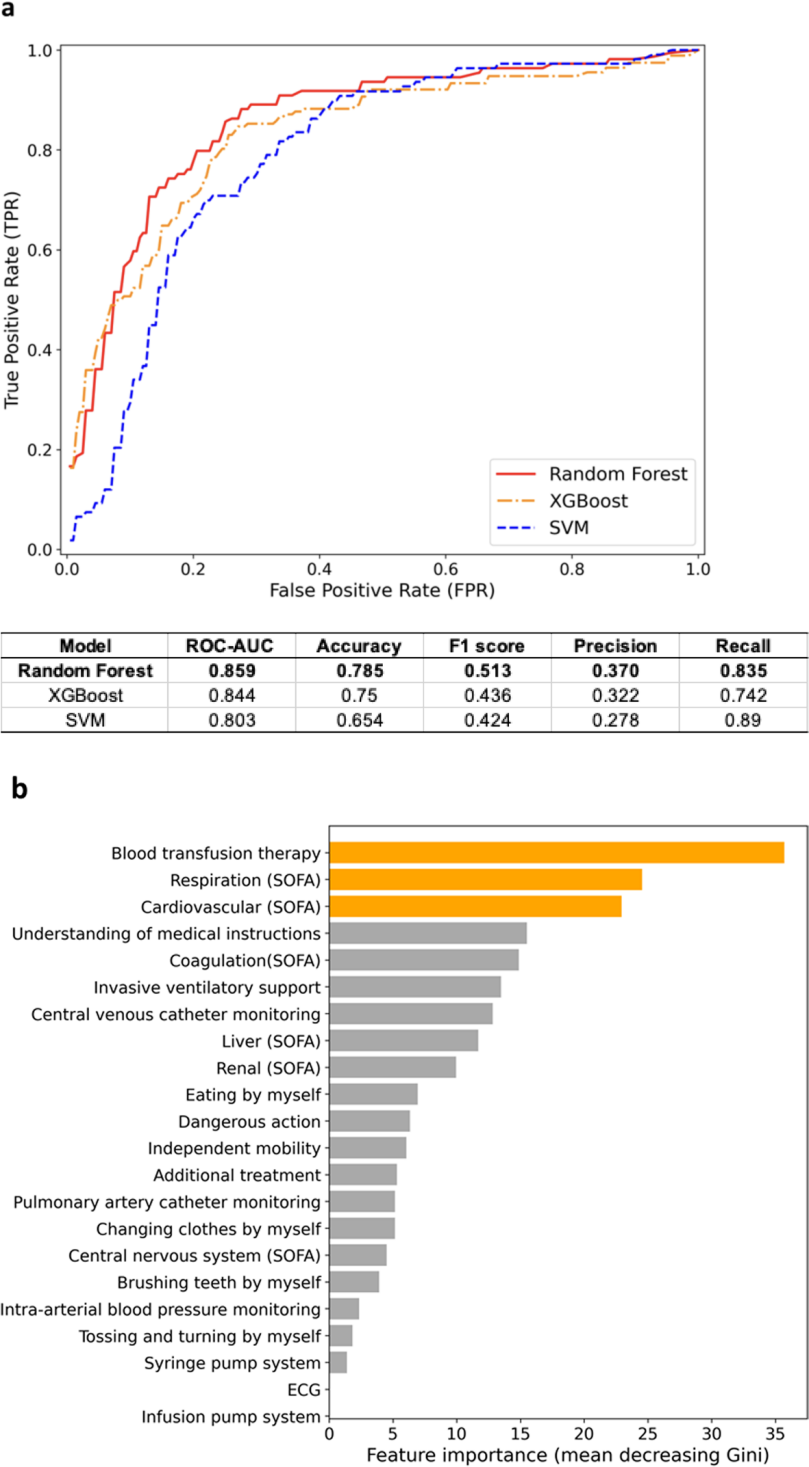
Predictive accuracy and feature importance for identifying a large negative contribution margin among postsurgical patients. **a** ROC curves and performance metrics (AUC, accuracy, precision, recall, and F1 score) were obtained from machine-learning methods: random forest, eXtreme Gradient Boosting, and support vector machine. **b** Relative importance of features for whether the negative contribution margin is large in random forest. Blood transfusion therapy, respiration (SOFA score), and cardiovascular (SOFA score) had the highest importance for precise prediction of whether the negative contribution margin is large. *ROC*, receiver operating characteristic; *AUC*, area under the curve; *ICU*, intensive care unit; *SOFA*, Sequential Organ Failure Assessment; *ECG*, electrocardiogram

The entire dataset of the 6743 patients was randomly split into training (4718, 70%) and test (2025, 30%) datasets. After random undersampling of the training set, 304 patients with financial loss events and 304 patients without events were retained, resulting in a balanced training cohort of 608 patients used for model fitting. Initially, the predictive values of the prediction model using the three machine-learning approaches were compared. RF had the highest predictive value for whether there was a negative contribution margin among the test dataset (Fig. 3a, red line; AUC, 0.859; accuracy, 0.785; precision, 0.370; recall, 0.835; and F1 score, 0.513). In the feature importance analysis using the RF model, blood transfusion therapy and respiratory and cardiovascular SOFA subscores were identified as important features (Fig. 3b).

### Sensitivity analysis for missing SOFA scores

In the sensitivity analysis including patients with missing or unclear SOFA scores, mean imputation was applied to replace missing values. This analysis comprised 8665 patients, of whom 527 (6.1%) experienced financial loss events. The 1922 patients who had been excluded in the main analysis due to missing or unclear SOFA scores were thus retained. The model demonstrated a similar overall discriminative ability compared to the complete case analysis (ROC-AUC, 0.841 vs. 0.859), although predictive performance for positive cases was slightly reduced (Additional file 2: Table 2).

## Discussion

Despite the possibility of financial deficits, intensive use of ICU resources is essential to improve clinical outcomes. From a hospital management perspective, accurately predicting the likelihood of patients to incur a large negative contribution margin would allow for the reallocation of medical resources and implementation of appropriate strategies for other patients without a financial deficit, thereby contributing to stable hospital operations. This machine-learning model, which predicts whether a large negative contribution margin will occur, based on clinical information on the first day of ICU admission, could be fundamental data for hospital management analysis.

This model was developed using DPC data from a single tertiary care hospital. While this limits generalizability, the methodology is scalable to datasets from other institutions or national claims, enabling broader applicability. Such models could serve as foundational tools in evaluating hospital operations and informing healthcare policy.

Feature importance analysis revealed that blood transfusion and respiratory and cardiovascular SOFA subscores were strong predictors of financial loss. The respiratory SOFA score reflects the need for invasive ventilatory support, which has been associated with increased ICU costs [20]. Similarly, cardiovascular SOFA scores often indicate hypotension requiring active interventions, which are also linked to increased mortality and resource utilization [21]. These findings are consistent with those of previous studies and support the validity of the model.

Conversely, variables related to patient monitoring or clinical status did not rank highly, likely because our cost outcome excluded labor costs—particularly those related to nursing, which account for about 30% of ICU expenditure [22]. While increased nurse staffing is associated with improved outcomes, these costs were not captured in our model, potentially underestimating the economic impact of nursing intensity.

This study also demonstrated that machine learning achieved high predictive performance in identifying postoperative patients in the ICU at risk of incurring a large negative contribution margin. Machine-learning techniques are increasingly used to predict healthcare costs based on administrative claims data due to their ability to process complex and large-scale information efficiently [23–27]. Previous studies have reported strong predictive performance for cost-related outcomes, with AUC values ranging from 0.74 to 0.776 [26, 27]. The predictive accuracy observed in our model was comparable to those reported in earlier studies. Furthermore, although the short median ICU stay in our cohort (1 [1-2] days) may limit the model’s direct clinical use at the bedside, it could be implemented to provide early warning flags for patients at high risk of financial loss upon ICU admission. Such information may support resource allocation, financial planning, and budget impact simulations, thereby contributing to hospital management.

This study has some limitations. First, it was a single-center, retrospective analysis, which inherently carries the risk of confounding and information bias. Furthermore, the generalizability of our findings may be limited, as the claims and reimbursement structure used in this study reflect the characteristics of a single institution. In addition, external validation was not performed, and the model was only evaluated on an internal test set. Future studies utilizing multicenter datasets are necessary to develop more generalizable machine-learning models that are not influenced by institutional variability.

Second, although cost accounting is the gold standard for estimating direct medical costs, it was not feasible in this study due to the large volume of data. As a result, we used an “estimated contribution margin” based on available claims data. Because medical fees are revised annually, this estimation may be subject to some inaccuracies, particularly regarding the pricing of services and supplies. Third, we excluded 1922 patients due to missing or unclear SOFA scores, introducing potential selection bias. However, this rate is consistent with national trends, and patients with missing scores had lower ventilator use and mortality, suggesting they were less severely ill [28]. To assess robustness, we conducted a sensitivity analysis retaining these patients with mean-imputed SOFA values. Discrimination was broadly similar to the primary analysis, but precision for positive cases was lower, which indicates that accurate SOFA scoring may contribute to predictive performance. Fourth, hyperparameter tuning was not performed in this study. This may limit the ability to directly compare the performance of different machine-learning models, and future studies with larger datasets should consider extensive hyperparameter optimization. Finally, to address class imbalance, we applied a single random undersampling of the majority class to achieve a 1:1 ratio in the training dataset. While this approach helped balance the classes and improve model fitting, it inevitably discarded a substantial portion of majority class data, which may have led to information loss and reduced stability due to dependence on a single random draw. The reliance on a single undersampling approach should therefore be considered when interpreting the stability and generalizability of the model.

## Conclusions

Machine-learning models can effectively predict the risk of a large negative contribution margin based on clinical information available on the first day of ICU admission. Among the predictive variables, SOFA scores and specific treatments, such as blood transfusion and ventilatory support, were key contributors to financial outcomes. Although the short median ICU stay may limit direct bedside application, our findings suggest that early prediction of financial deficits may support more informed reimbursement decisions and resource allocation, thus contributing to sustainable hospital management.

## Supplementary Information

Additional file 1: Table 1. Variables used in modeling.

Additional file 2: Table 2. Results of sensitivity analysis.

## Data Availability

The datasets used and analyzed during the current study are available from the corresponding author on reasonable request.

## Abbreviations

AUC: Area under the curve
DPC: Diagnosis procedure combination
DRG: Diagnosis-related group
ICU: Intensive care unit
RF: Random forest
ROC: Receiver operating characteristic
SOFA: Sequential Organ Failure Assessment
SVM: Support vector machine
XGBoost: Extreme Gradient Boosting
